# Mitigation of Biofilm Formation on Corrugated Cardboard Fresh Produce Packaging Surfaces Using a Novel Thiazolidinedione Derivative Integrated in Acrylic Emulsion Polymers

**DOI:** 10.3389/fmicb.2016.00159

**Published:** 2016-02-16

**Authors:** Michael Brandwein, Abed Al-Quntar, Hila Goldberg, Gregory Mosheyev, Moshe Goffer, Fulgencio Marin-Iniesta, Antonio López-Gómez, Doron Steinberg

**Affiliations:** ^1^Biofilm Research Laboratory, Faculty of Dental Medicine, Institute of Dental Sciences, The Hebrew University of JerusalemJerusalem, Israel; ^2^Institute for Drug Science, School of Pharmacy, The Hebrew University of JerusalemJerusalem, Israel; ^3^B. G. TechKibbutz Beit Guvrin, Israel; ^4^Food Technology, Nutrition and Bromatology Department, Facultad de Veterinaria, Universidad de MurciaMurcia, Spain; ^5^Department of Food Engineering and Agricultural Equipment, Technical University of CartagenaCartagena, Spain

**Keywords:** thiazolidinedione derivatives, food packaging, biofilms, *Pectobacterium carotovorum*, corrugated cardboard, food handling, food microbiology

## Abstract

Various surfaces associated with the storage and packing of food are known to harbor distinct bacterial pathogens. Conspicuously absent among the plethora of studies implicating food packaging materials and machinery is the study of corrugated cardboard packaging, the worldwide medium for transporting fresh produce. In this study, we observed the microbial communities of three different store-bought fruits and vegetables, along with their analog cardboard packaging using high throughput sequencing technology. We further developed an anti-biofilm polymer meant to coat corrugated cardboard surfaces and mediate bacterial biofilm growth on said surfaces. Integration of a novel thiazolidinedione derivative into the acrylic emulsion polymers was assessed using Energy Dispersive X-ray Spectrometry (EDS) analysis and surface topography was visualized and quantified on corrugated cardboard surfaces. Biofilm growth was measured using q-PCR targeting the gene encoding 16s rRNA. Additionally, architectural structure of the biofilm was observed using SEM. The uniform integration of the thiazolidinedione derivative TZD-6 was confirmed, and it was determined via q-PCR to reduce biofilm growth by ~80% on tested surfaces. A novel and effective method for reducing microbial load and preventing contamination on food packaging is thereby proposed.

## Introduction

Recent advances in next-generation sequencing (NGS) technologies has proven the existence of diverse and different bacterial communities on almost all surfaces known to man, including the human body, plants, soil, and water (Human Microbiome Project C, 2012; Kembel et al., 2014; Pinto et al., 2014; Panke-Buisse et al., 2015). This newfound understanding of the microbial world has shed light on the world of bacterial biofilms and food technologies, showing that different produce types harbor distinct bacterial communities and that fresh produce often serve as vehicles for the transmission of human pathogens, frequently by providing a suitable surface for biofilm growth (Berger et al., 2010; Critzer and Doyle, 2010; Leff and Fierer, 2013). Additionally, bacteria are often the causative agents of food spoilage, and can now be monitored, identified and potentially prevented using NGS technologies (de Boer et al., 2015).

Control and inhibition of bacterial biofilm formation in the food processing industry is of the utmost importance to both consumers and producers. Recently, attention has turned to anti-microbial coatings as a novel method for mitigating biofilm formation on food packaging materials. Polymers can be used to coat corrugated cardboard, nylon, plastic, and polystyrene used for food packaging. Engineering polymers with anti-adhesion chemical properties or integrating anti-microbials into active packaging are two common strategies being studied (Appendini and Hotchkiss, 2002; Zodrow et al., 2012). Additionally, other active ingredients for incorporation into active packaging have been studied, including organic acids, enzymes, bacteriocins, fungicides, natural extracts including essential oils, ions, ethanol, etc. (Ramos et al., 2013). However, to the best of our knowledge, synthetic molecules meant to mitigate biofilm growth have yet to be deployed in anti-microbial/anti-biofilm polymers.

Thiazolidinedione analogs have been shown to be prospective biofilm inhibitors (Brackman et al., 2013,2014; Feldman et al., 2014; Kagan et al., 2014), and therefore are an attractive molecule to be included within a broadly applicable anti-biofilm polymer. They have been shown to reduce biofilm formation in the bacteria *Propionibacterium acnes* (Brackman et al., 2014), *Pseudomonas aeruginosa* (Lidor et al., 2015), and the yeast *Candida albicans* (Feldman et al., 2014; Kagan et al., 2014).

*Pectobacterium carotovorum* subsp. *carotovorum*, formerly *Erwinia carotovora* subsp. *carotovora*, is one of the causative agents of potato tuber soft rot and blackleg, which causes severe economic loss worldwide (de Haan et al., 2008). Its virulence is not restricted to potatoes, and can affect carrots, lettuce, and peppers, amongst others (Tsuchiya et al., 1979; Coplin, 1980; Hassenberg et al., 2008). They produce cell wall degrading enzymes, which enables for the infection of the host. Once infected, the organism feeds on the host tissue, further degrading its quality (Czajkowski et al., 2011). It has been posited that a critical virulence factor for the pathogenesis of the *P. carotovorum* is the production and secretion of homoserine-lactones, a known quorum sensing compound, to further enable the development of the biofilm (Barras et al., 1994). Thus, biofilm formation is a critical step in the formation of soft rot by *P. carotovorum*.

We aim to assess the relatedness of bacterial communities found on several fresh fruits and vegetables and their associated corrugated cardboard surfaces and then the feasibility of mitigating biofilm formation on a broad array of surfaces by developing a novel polymer supplemented with thiazolidinedione type anti-biofilm molecules.

## Materials and methods

### Sample collection and DNA extraction

Clementines, tomatoes, and sweet potatoes and their corrugated cardboard packaging analogs were collected from two different grocery stores in Jerusalem, Israel on December 18, 2014. The samples were immediately transferred to the laboratory in sterile plastic bags, and swabbed with a sterile cotton swab soaked in 0.15 M NaCl and 0.1% Tween 20 (Gao et al., 2010). DNA was extracted using the MoBio Powersoil DNA Extraction Kit following the manufacturer's instructions. Primers targeting the V3 and V4 regions of the bacterial 16s gene were used for the PCR step (Klindworth et al., 2013). Amplicon PCR, index PCR, and PCR cleanup steps were carried out following Illumina's published 16s metagenomic sequencing library preparation protocol. Libraries were quantified and normalized using Qubit and sequenced on a MiSeq sequencer. Data analysis was performed using Illumina's Basespace 16s Metagenomics software and QIIME (Caporaso et al., 2010).

### Topography characterization using a 3-D profilometer

3-D topography characterization was performed as described previously (Mousavi et al., 2014). Briefly, a 3-D profilometer (Bruker Contour GT-K1, Germany), was used to study the roughness and the morphology of the corrugated cardboard and polymer-covered corrugated cardboard surfaces. This equipment uses light interferometry with a 50 × objective to capture surface roughness in increments ranging from 130 nm to 1 mm. The Contour GT-K1 is delivered with a dual-LED light source, a focus module controlled by computer and a measure table that can be tilted or moved to ensure a greater precision and allow more sample geometries. The tests were performed using the VSI (vertical scanning interferometry) technique along with the remove 8 tilt filter that compares every point to its neighbors and provides a 3D picture of the sample free of tilt influence.

### Synthesis of TZD-6 and incorporation in polymers

Thiazolidinedione derivative TZD-6 (**Figure 3A**) was synthesized according to a previously described method with some modifications (Feldman et al., 2014). Briefly, 0.085 g (1 mmol) piperidine was added to 0.117 g (1 mmol) thiazolidine-2,4-dione in 8 mL ethanol, followed by the addition of 0.090 g (0.9 mmol) of hexanal. The mixture was stirred for 30 min at room temperature and was then refluxed in ethanol for 24 h. After cooling to 0°C, 5 mL of 1 M HCl solution was added to the mixtures and it was maintained at 4°C for several days. The precipitate was subsequently filtered, washed with petroleum ether, dried, and analyzed by NMR, melting point and elemental analysis, amounting to a 60% yield with chemical purity above 95%. Polymers used in this study were produced by B.G. Tech, of Kibbutz Beit Guvrin in Israel and are pure acrylic co-polymers and are water-based emulsions. No solvents are used in the production process, rather surface active agents including anions and nonions are used. After preparation, TZD-6 molecules were dissolved in acrylic emulsion polymer, to a final sub-minimal inhibitory concentration (MIC) of 0.42% (w/w). Integration of TZD-6, a powder-like hydrophobic substance, into water-based polymers requires an initial dissolving step using an amine-based dissolving agent, 95% 2-amino-2-methyl-1-propanol solution. This solution was diluted 1:10 and TZD-6 was then dissolved in it. The mixture was then added to the polymer and no powdery substance was visible with either the naked eye or through SEM (micrograph not shown). Coatings were applied to polystyrene pegs by placing pegs in wells filled with polymer, and removing to dry. Polymers were allowed to dry for 24 h.

### Energy dispersive X-ray spectrometry (EDS) analysis

The ability of the TZD-6 to disperse throughout the final coating of dry polymer mixture was assessed using EDS analysis. The method measured the relative amount of sulfur, a unique constituent of the TZD-6 molecule, in the overall polymer mixture. Polymer infused with 3.6% (w/w) TZD-6 was used to coat glass cover slips (Thermo Scientific) and was analyzed with a scanning electron microscope equipped with EDS (Quanta-200, FEI). Three different samples were examined; polymer without TZD-6 (control), polymer with 4% TZD-6, and polymer with 4% TZD-6 (same concentration) which was inverted and left to dry. In each sample, four regions were analyzed for the percentage chemical composition of the polymer surface using an electron beam 5 μm in diameter and an X-ray detector system attached to the SEM. The beam analyzed the uppermost 5 μm of polymer, whereas polymer thickness, or depth to glass surface, was 300 μm. This allowed for characterization of the polymer surface alone. The method allowed relative amounts of carbon (C), oxygen (O), and sulfur (S) to be determined. Relative amounts of sulfur were used to determine uniform dispersal of TZD-6 in polymer.

### Bacterial strains and culture conditions

*P. carotovorum* subs. *carotovorum* strain PC1, kindly provided by Dr. Iris Yedidia of the Institute of Plant Sciences, Agricultural Research Organization, Volcani Center, was stored in glycerol stock at −80°C and was revived and incubated overnight in LB Broth at 30°C.

### Biofilm formation

Microtitre wells (96W, Nunc) were inoculated with 200 μL of the standardized bacterial culture described above and immediately covered with polymer-covered pegs. The plates were then incubated for 24 h at 37°C to allow biofilm formation. Following incubation, pegs were gently washed with saline (Herrmann et al., 2010).

### qPCR quantification of bacteria in biofilm

DNA was extracted from biofilm using a modified alkaline lysis protocol as previously described (Periasamy and Kolenbrander, 2009). Briefly, DNA was extracted in 0.05 M sodium hydroxide for 60 mins at 60°C and the pH was subsequently neutralized with the addition of 1 M Tris-HCl (pH 7.0). Extracted DNA was stored in −20°C and subsequently used as a template for qPCR analyses. 16s universal primers 5′TCCTACGGGAGGCAGCAGT-3′, and 5′GGACTACCAGGGTATCTAATCCTGTT-3′ were used (McIntosh and Hajishengallis, 2012) and SYBR green dye (Invitrogen) was added to detect amplicons. qPCR reaction was carried out in an ABI prism instrument (Applied Biosystems Prism 7300). A standard curve was constructed using DNA extracted with a GenElute Bacterial Genomic DNA kit (Sigma-Aldrich) from an overnight culture of *P. carotovorum*. Three samples were used: corrugated cardboard (control), bare polymer covered corrugated cardboard, and polymer with TZD6 applied on corrugated cardboard.

### Scanning electron microscopy

A modified method was used to analyze and visualize biofilm formed on polymer-covered corrugated cardboard. Following 48 h incubation, corrugated cardboard discs were transferred from agar plates and washed gently in saline. Twenty-five microliters of 4% formaldehyde was applied to fixate the cells. Incubation commenced at room temperature for 30 min and the discs were again washed in saline. The discs were then incubated at room temperature in a laminar flow hood for 2 h to allow for complete drying of the sample. Samples were then mounted on a metal stub and sputter coated with gold prior to analysis by SEM.

### Statistical analysis

Statistical analysis was performed using a student's *T*-Test on Microsoft Excel to compare bare polymer and polymer + TZD-6 surfaces. Statistical significance was determined as a *P*-value below 0.05.

## Results

### Microbial community analysis of clementines, sweet potatoes, tomatoes, and their analog corrugated cardboard surfaces

**Figure 1:**
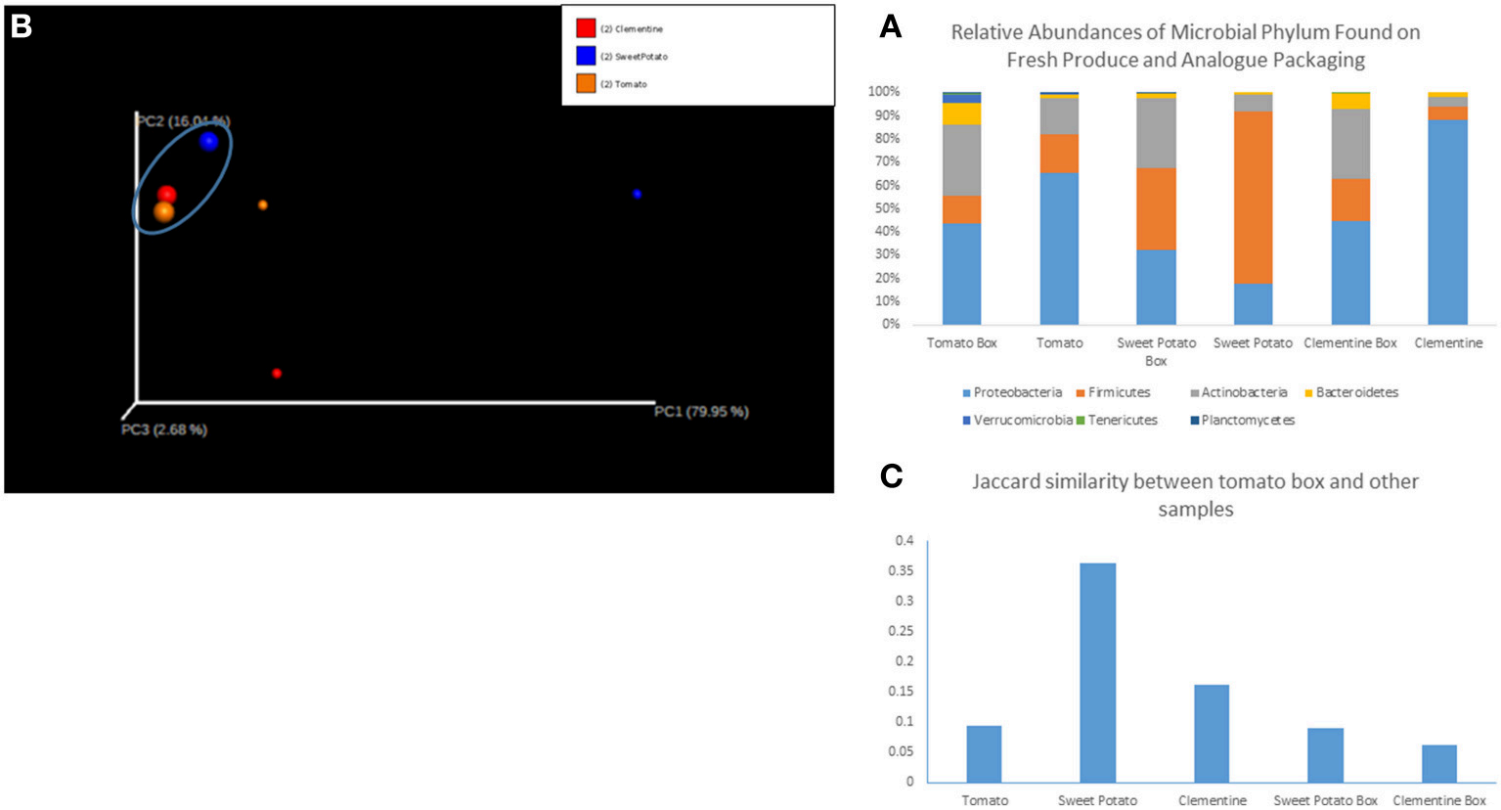
**(A)** Relative abundances of microbial phylum found on fresh produce and analog packaging. **(B)** Principal coordinates analysis of samples. The cluster of samples in the circle at the top left (larger circles) represent corrugated cardboard associated samples. Red circles represent clementine and analog box, blue represents sweet potato and analog box, and orange represents tomato and analog box. **(C)** Jaccard similarity between tomato corrugated cardboard and other samples.

We sequenced six samples on an Illumina MiSeq and obtained 1.4 million reads that passed Illumina's quality filters, of which 88% were classified to a representative genus. Sequences that mapped to the phylum Cloroflexi and Cyanobacteria (phylum level analysis) and to the genus *Calothrix* (genus level analysis), a cyanobacteria, were removed as they were assumed to represent the chloroplast sequences from the produce, and not resident microorganisms. Shannon diversity index (data not shown) of the samples did not show any significant trends. Relative abundances of each sample, grouped by fruit/vegetable and its analog packaging show distinct microbial communities in each sample type, with each sample harboring 6–7 dominant phyla (Figure 1A) and ~15–25 dominant genera. Proteobacteria, Firmicutes and Actinobacter comprise roughly 90% of the bacterial phyla in each sample. However, the less abundant phyla, Bacteroidetes, Verrucomicrobia, Terenicutes, and Plantomycetes, are all consistently present in all samples. Beta diversity, as measured by the binary Jaccard index (BJI), was used to generate a PCoA plot (Figure 1B), which shows the clustering of microbial communities associated with corrugated cardboard (large dots). Conversely, microbial communities from the surface of the clementine, sweet potato, and tomato were all distinct, primarily along the PC1 axis. The BJI also indicated relative similarity between the tomato box and its tomato analog, as well as between the tomato box and the other two boxes (Figure 1C).

### Surface topography smoothing with application of polymer

**Figure 2:**
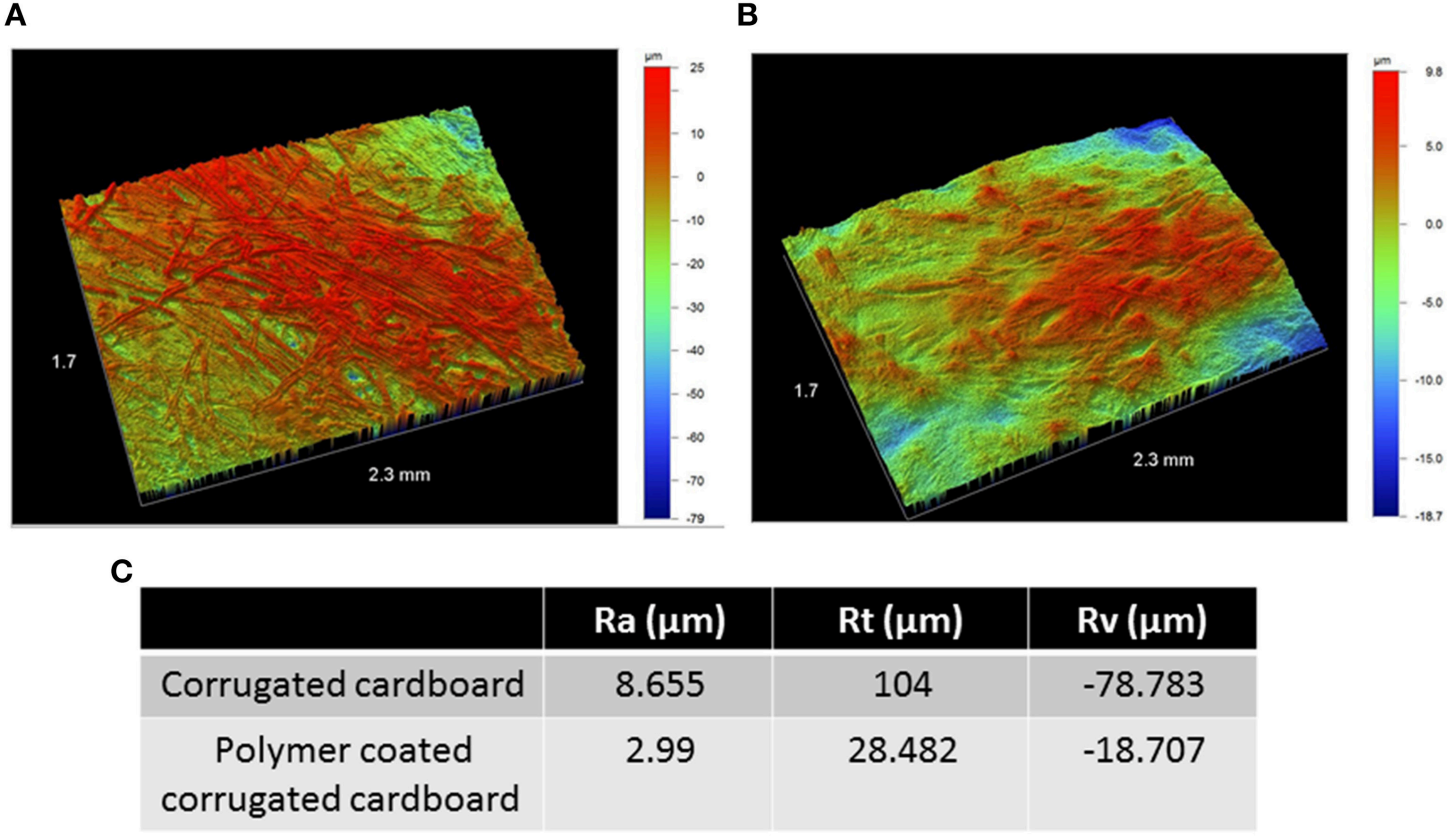
**Surface topography of bare corrugated cardboard surfaces (A) and polymer coated surfaces (B)**. Color scale to right of pictures denotes height and depth in microns. Columns in table: Profile roughness parameter (Ra), the maximum height (Rt), and maximum valley depth (Rv) of uncoated corrugated cardboard surfaces (row 1) and coated (300 μm) surfaces (row 2) **(C)**. Both pictures and table indicate tempering of roughness profile with the addition of polymer on cardboard.

We analyzed the effect of polymer application on corrugated cardboard topography using an optical profilometer, which allowed us to measure the profile roughness parameter (Ra), the maximum height (Rt), and maximum valley depth (Rv) of uncoated corrugated cardboard surfaces and coated (300 μm) surfaces. All three parameters were significantly less extreme with the application of polymer on corrugated cardboard (Figure 2), effectively tampering the roughness profile and smoothing the surface.

### TZD-6's incorporation into polymer

**Figure 3:**
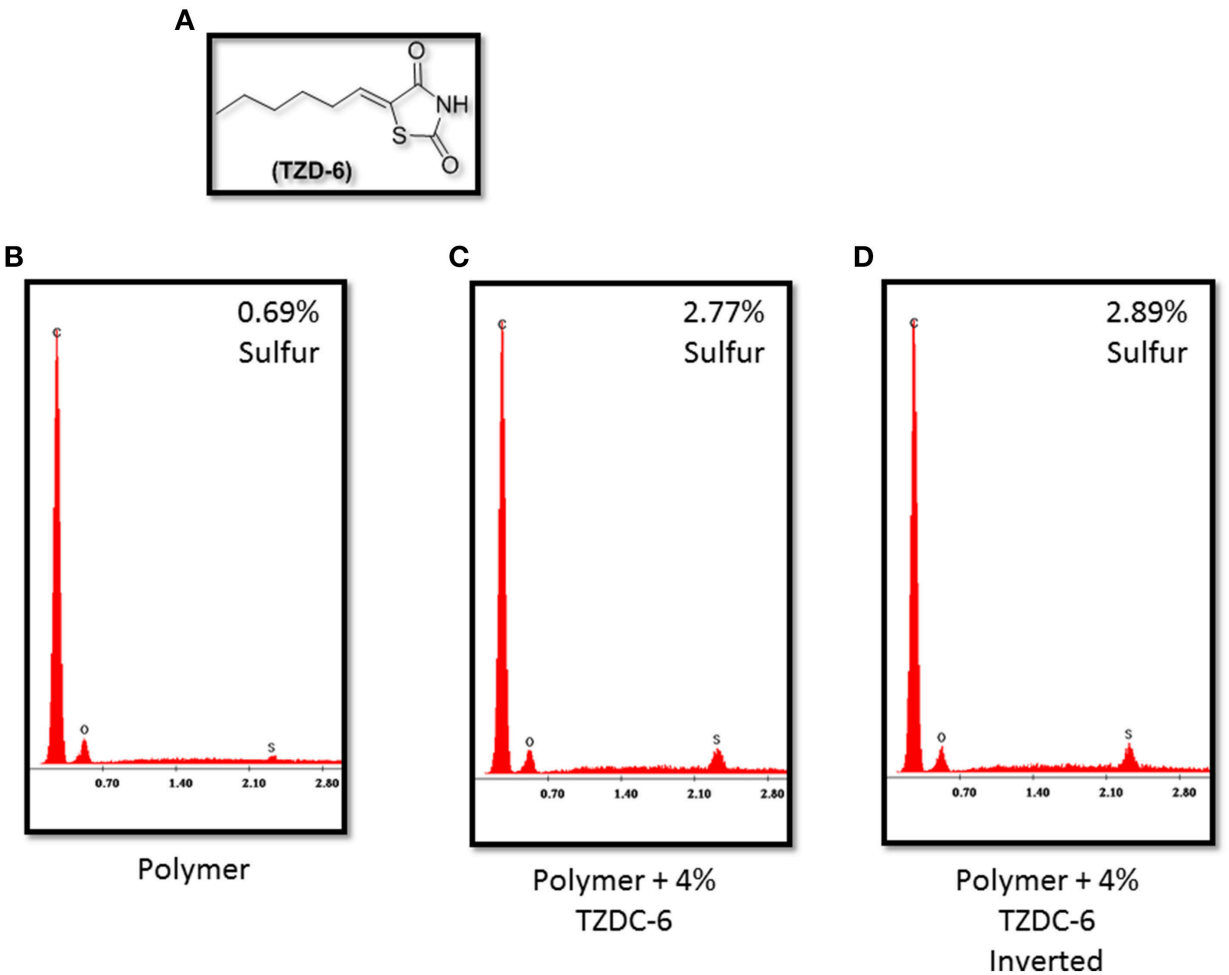
**(A)** Thiazolidinedione derivative TZDC-6. Energy Dispersive X-ray Spectrometry graphs indicating relative sulfur concentrations in TZDC-6 infused polymers. Naked polymer **(B)** shows <0.69% sulfur composition, while both samples coated with TZDC-6 infused polymers **(C,D)** show similar TZDC-6 concentrations on surface, irregardless of drying angle. Percentage values represent a mean of four different isolated points tested. C, Carbon; O, Oxygen; S, Sulfur.

Figure 3B verifies that no significant traces of sulfur are detected on the surface of the pure acrylic emulsion polymer. However, both samples, each left to dry at opposite angles, infused with 4% (w/w) TZD-6 indicate almost the same sulfur surface composition, independent of drying angle (Figures 3C,D). The aforementioned data, coupled with SEM observations led us to conclude that our method of a step-wise amine-based dissolving process of the TZD-6 produced a homogenous polymer-TZD-6 mixture. This conclusion, coupled with the previous observation regarding surface smoothing, propelled our research into the biofilm preventing properties of our anti-biofilm polymer.

### Biofilm mitigation of polymer/TZD combination

**Figure 4:**
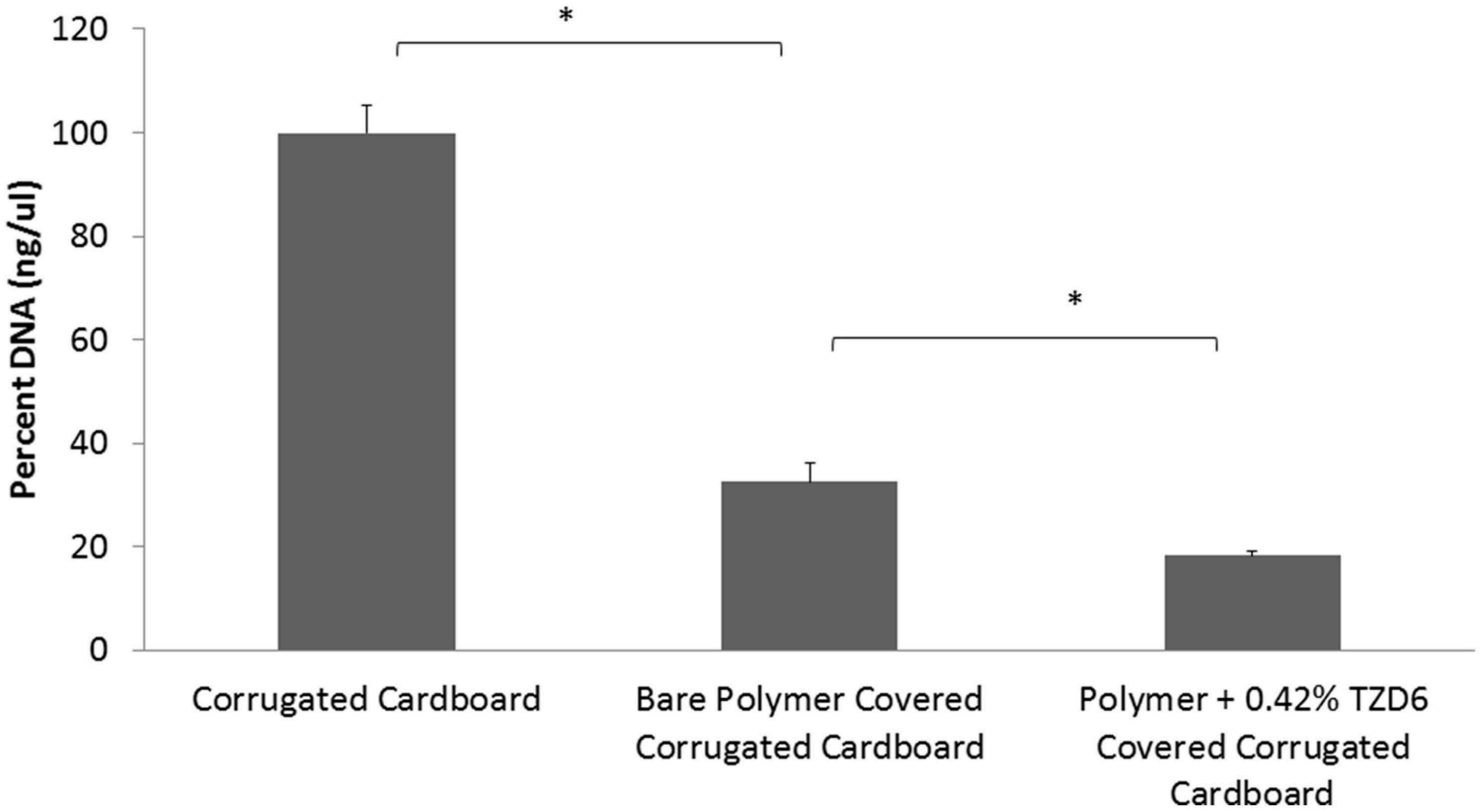
**qPCR quantification of 16s DNA of lysed *P. carotovorum* biofilms grown on corrugated cardboard discs**. Application of bare polymer on corrugated cardboard surfaces leads to a 67.5% reduction in biofilm. Incorporation of TZDC-6 into the polymer results in an additional 14.3% reduction when compared to uncoated surfaces, and 43.9% when compared to bare polymer covered surfaces. ^*^P < 0.05.

**Figure 5:**
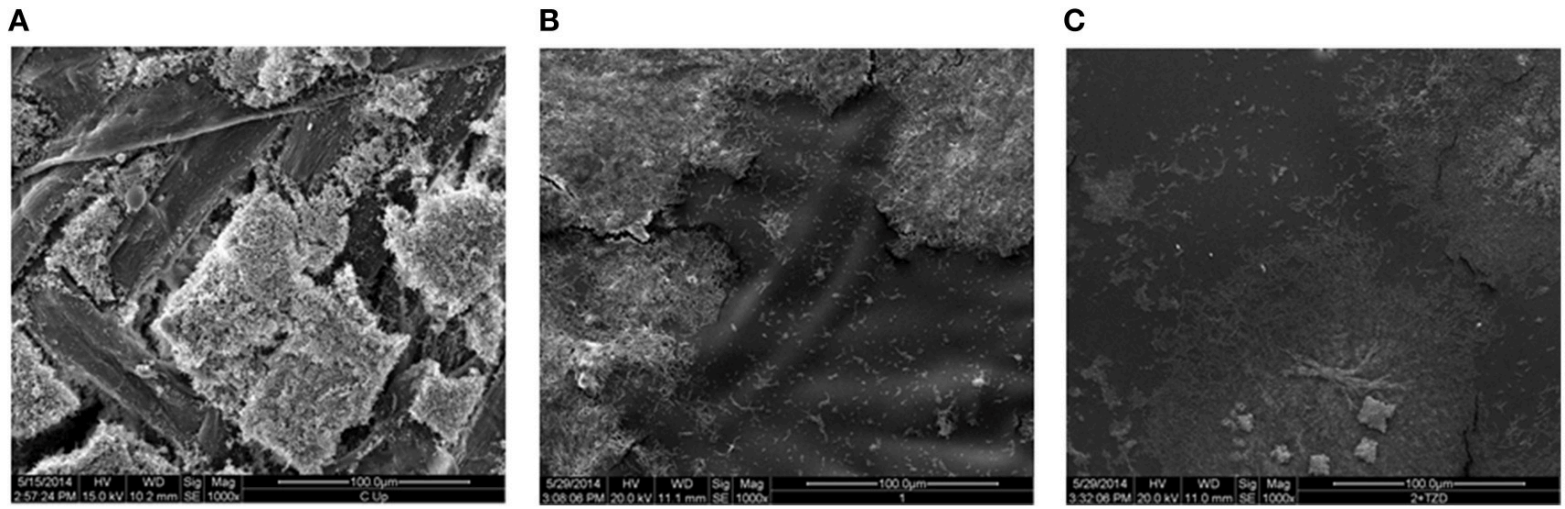
**Scanning electron micrographs of *P. carotovorum* biofilms grown on (A) corrugated cardboard surfaces without polymer, bare polymer covered corrugated cardboard (B), and polymer + TZD-6 covered corrugated cardboard (C)**. Cellulose fibers are clearly discernible on the bare corrugated cardboard images, as are robust biofilms. Images of coated surfaces show significantly less biofilm (white rods/clusters). This effect is magnified with the introduction of TZD-6 into the polymers **(C)**.

A qPCR assay on described surfaces with *P. carotovorum* biofilm growth (Figure 4) confirmed biofilm mitigation of bare polymer and additional biofilm mitigation with the addition of TZD-6 into the polymer. Biofilm decreased by 67.5% with the simple application of polymer bereft of TZD6, and a further 14.3% reduction was observed with the incorporation of TZD6 into the polymer (*P* < 0.05). Both reductions were deemed significant based on a student's *T*-test as described above. Micrograph images of *P. carotovorum* biofilm shows complete infestation of bare corrugated cardboard throughout the entire depth and breadth of the disc. Marked reduction of bacterial load is observed with the application of bare polymer on the cardboard, as can be seen vividly by comparing Figures 5A,B. Furthermore, the introduction of TZD6 into the polymer (Figure 5C) leads to a further diminishment of biofilm.

## Discussion

This study represents a first look at the relationship between corrugated cardboard packaging and fresh produce. The curious lack of information regarding the microbial consequences of such a packaging system stands out in stark contrast to other packaging systems, which have been studied in depth (Caleb et al., 2013; Jideani and Vogt, 2015). The worldwide increase in fruit and vegetable consumption, coupled with the concurrent increase in foodborne illnesses as a result of consuming such produce, requires effective, and novel strategies to alleviate the public health concern (Olaimat and Holley, 2012). Here, we demonstrate the co-carriage of microorganisms by both fresh produce and their correlated packaging material. While Proteobacteria was observed to be the dominant phylum in the tomato, tomato box, clementine and clementine box samples, Firmicutes proved to be the leading phylum in sweet potato and sweet potato box samples. A similar shift is observed between the tomato and its packaging equivalent and between the clementine and its packaging. This shift is typified by the reduction in the relative abundance of Proteobacteria along with the concurrent increase in the relative abundance of Actinobacteria. As a whole unit, the corrugated cardboard surfaces were more similar to one another than to their corresponding fruit or vegetable surfaces. This observation posits a selection of microorganisms suitable for living on cardboard surfaces being transferred from the fresh produce to the packaging. Additionally, the surfaces of the clementine, tomato, and sweet potato were markedly different, indicating a unique and distinct microbiome for each produce type. Such an understanding of the diverse nature of bacterial communities associated with the surfaces of fresh produce is in line with recent reports (Leff and Fierer, 2013). Finally, the tomato and tomato packaging harbored the most similar microbial communities among the produce-packaging sets. These understandings point to two important conclusions for the study of food-microbe relationships; (a) Certain packaging types can select for, and potentially enrich, certain microbial community members and (b) Certain produce types may harbor similar microbiomes to their packaging analogs. Either way, it would be judicious to prevent microbial proliferation both on the surfaces of fresh produce and on its packaging in the time they are in contact from harvest to market.

The study of biofilm prevention on surfaces and through polymers has proven bountiful, yet such an application on corrugated cardboard surfaces has yet to surface. Given the conclusion that corrugated cardboard packaging does indeed harbor microbial communities, we aimed to develop a polymer infused with anti-biofilm molecules to mitigate biofilm growth on such surfaces. However, before testing the biological activity of such a polymer, we characterized the ability of such a polymer to alter the physical landscape of the cardboard surface. Surface roughness may be a critical factor in encouraging bacterial growth on corrugated cardboard cellulose fibers, as it has been noted in other experimental systems including PMMA, stainless steel and titanium (Gad et al., 2012). Indeed, we showed that application of polymer devoid of any TZD-6 both reduced surface roughness and significantly tempered the peaks and troughs (Figure 2). Such an environment is theoretically less hospital for bacteria, as it provides for a less nuanced surface. Additionally, a recent study noted that several Salmonella strains attached and grew in significantly larger quantities than *L. monocytogenes* on cellulose surfaces (Tan et al., 2013), indicating that cellulose surfaces can be particularly good environments for certain pathogens.

Once we had established the significant changes in surface topography the polymer could effect, we set out to incorporate a novel thiazolidinedione analog in our polymer. Thiazolidinedione analogs have been shown to be biofilm inhibitors in *P. acnes, C. albicans* and *Vibrio harveyi* (Brackman et al., 2013,2014; Feldman et al., 2014; Kagan et al., 2014; Lidor et al., 2015). Given the novel approach of incorporating potential quorum sensing molecules in polymers, we aimed to assess the uniform dispersal of such agents in the polymer, thereby ensuring consistent coverage both over the polymer's surface and throughout its depth. The Energy Dispersive X-ray Spectrometry (EDS) Analysis results validate the successful uniform integration of TZD-6 into the polymer and a SEM micrograph of the polymer with TZD-6 displays an even and homogenous coating of the surface (SEM micrograph not shown). Such knowledge validates the use of the polymer as a vehicle for delivery of the TZD-6. Uniform and even dispersal allows for precise calculations of the ratio of active agent to target in a product.

Biological assays verified that TZD-6 is still biologically active after the incorporation into the polymeric matrix and inhibits biofilm formation (Figures 4, 5). qPCR quantification of 16s DNA showed that application of bare polymer on corrugated cardboard surfaces mitigated over half of the bacteria constituting the *P. carotovorum* biofilm. Additionally, when comparing cardboard coated with bare polymer to that covered with polymer infused with TZD-6, we show that the TZD-6 effects an additional ~50% decrease in bacterial load. Taking the two together, over 80% of the biofilm is eradicated with the application of our anti-biofilm polymer on corrugated cardboard packaging materials. Traditional methods for quantifying biofilm biomass and depth (Francolini et al., 2004), such as crystal violet staining and confocal laser scanning microscopy were unemployable in our project, as the polymer contributed signals stronger than the densest of biofilms. However, SEM provided us with micrographs that corroborated the previous qPCR results, showing sequential depletion of the biofilm first with the application of bare polymer and then with the application of polymer infused with TZD-6.

It has been posited that thiazolidinedione derivatives can serve as quorum sensing inhibitors in the AI-2 signaling pathway, thereby inhibiting biofilm growth (Brackman et al., 2013,2014; Feldman et al., 2014; Kagan et al., 2014). Given the widespread occurrence of food-borne diseases associated with microorganisms, food packaging has been noted as an attractive target for active anti-microbial and/or bacteriostatic packaging (Padgett et al., 1998; Lui et al., 2013). By incorporating only sub-MIC concentrations of the active agent, minimal amounts are introduced into the packaging, thereby leading to a cost-efficient method for preventing biofilm formation. While several studies mentioned elsewhere in this article have researched thiazolidinedione type molecules and their effect on biofilm formation, none have done so in the context of incorporation of the molecules into polymeric matrixes. Consequently, we explored many different methods of incorporating TZD-6 into various polymers, with varying results. Chief among our challenges was the hydrophobic nature of TZD-6 and the preference toward water-based polymers in the corrugated cardboard food packaging industry.

*P. corotovorum*, although non-pathogenic for humans, serves a disrupting role in the post harvest handling of fresh produce. By targeting organisms responsible for food loss, of which *P. carotovorum* is a prime culprit, we can effectively extend the shelf life of produce otherwise wasted (Tournas, 2005). Additionally, thiazolidinedione type molecules have been shown to mitigate biofilm formation in a wide variety of microorganisms. We hope to assess the anti-biofilm properties of TZD-6 on other food-associated bacteria in the future. Such bacteria may be causative agents of food loss or human pathogens. Recent studies have pointed toward vegetables as potential reservoirs for human pathogens, thereby further strengthening the need to control bacterial community structure on both food and its packaging (Berg et al., 2014).

Roughly 9 million tons of corrugated cardboard is produced annually worldwide for use as a post-harvest transport medium for fresh produce. Bacteria have the ability to propagate in the time that the agricultural yield spends between harvest and consumption, thereby either causing damage to the host produce or potentially to the final consumer (Eckert and Ogawa, 1988). With this knowledge in hand, the impetus to control microbial growth on corrugated cardboard takes on newer urgency. We postulate that corrugated cardboard serves as a particularly thriving environment for bacteria. This can be due to its specific surface adhesion properties, its vast network of cellulose fibers which create a complex three dimensional environment with increased surface area, and/or other physicochemical interactions. We suggest undertaking a more robust characterization of additional produce types, their analog packaging systems and both of their microbiomes. Such a study should document both spatial and temporal effects, much like a recent study that explored the lettuce leaf resident microbiota (Rastogi et al., 2012).

We present a novel and effective method of high potential for controlling bacterial biofilm growth on surfaces, specifically food packaging. The successful integration of anti-biofilm molecules in industry compatible polymers provides a cost-efficient solution to the health risks associated with consuming fresh produce. The use of antibiofilm agents as prescribed above can drastically reduce the occurrence of food-borne microbial human pathogens as well as bacteria and fungi associated with food spoilage. We are conducting further tests to validate the anti-biofilm effects of the above prescribed packaging systems for use with fresh fruit, vegetable, and meat products.

## Author contributions

MB performed all of the experimental work and wrote the manuscript. AA synthesized and purified the TZD molecules. HG, GM, and MG synthesized and supplied the polymer. DS initiated and coordinated the study, with valuable input from FM and AL. All authors reviewed the manuscript. All authors read and approved the final manuscript.

### Conflict of interest statement

The authors declare that the research was conducted in the absence of any commercial or financial relationships that could be construed as a potential conflict of interest.
